# Is Depression “Evolutionary” or Just “Adaptive”? A Comment

**DOI:** 10.1155/2010/631502

**Published:** 2010-03-08

**Authors:** Christopher F. Sharpley, Vicki Bitsika

**Affiliations:** ^1^Brain & Behaviour Research Group, University of New England, New South Wales, P.O. Box 378, Coolangatta, Qld, 4225, Australia; ^2^Brain & Behaviour Research Group, Bond University, Queensland, Australia

## Abstract

Some recent explanations of depression have suggested that it may be “evolutionary” in that there are advantages to the depressed individual which arise from some aspects of depressive symptomatology. While the depressive behaviour of withdrawal from the adverse environment may provide some immediate benefits to the depressed individual, thus making it potentially “adaptive” in the short-term, this does not fit the biological definition of “evolutionary”. In fact, depression does not meet two of the three required criteria from natural selection in order to be evolutionary. Therefore, while some depressive behaviour may be advantageous for the depressed individual, and is therefore “adaptive” in an immediate sense, it cannot be accurately described as “evolutionary”. Implications for research and clinical practice are discussed.

## 1. Genes, Behaviour and Depression

Clinical and subsyndromal depression adversely affect physical health, relationships, and cognitive performance [1–4]. With between 13% (Europe) and 17% (USA) of people having a major depressive episode at some time in their lives [5–7], depression has been described as the major contributor to the total disease burden [8] and predicted to become the second leading cause of mental illness by 2020 [9, 10]. As an indicator of its effect upon health, depression has recently been shown to have almost the same predictive power for mortality as does smoking [11]. One of the strongest predictors of depression is stress in the form of demanding challenges across a range of areas [12], acting through a variety of causal pathways including genetic and behavioural factors which may undermine an individual's ability to withstand stress [13, 14]. 

Although genetic linkage studies in depression have not previously been as successful as those for other disorders such as schizophrenia [15], there are some recent findings regarding the genetic basis of depression which have suggested specific genes which may, when combined with perceived uncontrollable environmental challenges, have statistically significant relationships with major depressive disorder (MDD). For example, Wang et al. [16] identified four genes that activate the secretion of corticotrophin-releasing hormone (CRH) from the hypothalamus and that show significantly higher expression in depressed than in nondepressed persons. CRH and its downstream consequent cortisol are consistently elevated in depressed patients [17–20], and these elevations have been associated with neuronal apoptosis in the hippocampus and prefrontal cortex and increased neurogenesis within the amygdala, leading to what Arnsten [21] referred to as a change from cognitive-based “top down” to emotion-based “bottom up” decision-making during chronic stress, which is a characteristic of depressed persons. Other genetic links with depression have been reported through polymorphisms and allele dominance on the serotonin transporter gene *5-HTT*, [22], the piccolo gene *PCLO,* which influences monoaminergic neurotransmitters in the brain [23], and gene-linked malfunctions of the glutamate and GABA systems [24], which reduce the functionality of the neurotransmitters serotonin [25] and dopamine [26], thus impairing cognitive performance, one of the symptoms of depression [27]. A recent meta-analysis of 193 research reports on 393 genetic polymorphisms identified six “MDD susceptibility genes” [14, page 772] that showed statistically significant relationships with MDD. Perhaps highlighting the “active” role of the gene-environmental interaction hypothesis of depression [28], Belsky et al., [29] has suggested that instead of considering some genes as representing a vulnerability to depression, they might more accurately be described as “plasticity genes” (page 746) because they predispose some individuals to depressive responses to environmental stressors. 

Following from the initial genetic predisposition or vulnerability to depression, behaviours that have been associated with depression include negative self-evaluation, depressive attributional style, distorted cognitive processing, deficiencies in social skills, avoidance behaviours [30] and ineffective problem-solving [31], some of which may contribute to the depressed person's perception that a particular environmental stressor is uncontrollable. While these factors exist in their own right as research-based predictors of depression, they more accurately should be seen as the “behavioural pathways” through which an individual may respond to a major environmental stressor. As will be described in detail below, those pathways can be subsumed under the general descriptor of “active withdrawal” from stressors that are perceived as inescapable and uncontrollable [32]. These behavioural factors may be more likely to occur as a result of genetic predisposition, but they usually require the learning of behaviour patterns from previous experience to become entrenched within an individual's behavioural repertoire. That is, individuals will respond to stressors according to their combined genetic and behavioural characteristics. Those responses may perhaps be influenced differentially (i.e., reinforced or extinguished) over time and circumstances by these characteristics and the relevant impact which a particular stressor has upon the individual. Evidence of variability in individuals' depression symptoms over time and/or depressive episodes points to such differential responsivity to stressors and supports the construct of an individual stressor-depressive symptoms interaction model [33].

## 2. Evolutionary or Adaptive?

### 2.1. Evolutionary

To explain the presence of those genetic-based factors which predispose certain individuals to depression, several papers have hypothesised an “evolutionary” model of depression which focuses upon the apparent benefit that emitting depressive behaviours in response to stressors has for the depressed individual (e.g., [34–41]). That literature suggests that depression has evolved to perform two problem-solving functions: (1) to withdraw from the stressor and so allow the depressed individual time to analyse the stressful situation they find themselves in, and (2) to elicit assistance from partners and close others [42]. However, this model is not without its critics (e.g., [43, 44]) and may also be misnamed on biological grounds. 

That is, from a biological perspective, for a trait (such as a predisposition to depressive behaviour) to be considered as “evolutionary”, it must fulfil several criteria. Following Darwin's [45] postulates and more recent developments [46, 47], different traits naturally exist between individuals within a population, and if those different traits are passed on to offspring *and* confer a survival or reproductive advantage to those offspring, the traits will be naturally selected and will become more common. When this happens, it may be said that evolutionary processes are at work [48]. That is, as a series of steps: (1) the trait in question must be present in one generation and passed on to the next generation via genetic inheritance; (2) the attribute that is passed on must confer some “selective fitness” benefit to the offspring of the parent with the trait [49, 50]). That is, the trait must pass from the parent to the offspring *and* must enhance the second generation's chances of reproducing successfully (defined as bringing their own offspring to reproduction age) [47, 51]. Of particular importance to the current discussion, the trait cannot be *developed* (e.g., in response to environmental stressors) during the parent's lifetime (that would be Lamarckism, not “evolutionary” in the current Darwinian sense). Following this model, to be an evolutionary-based behaviour, depression in parents would (a) somehow enhance their chances of reproducing successfully, (b) be passed on to their children, and (c) enhance their children's chances of also reproducing successfully. As will be explained below, only one of these three requirements is met by depression. 

Taking the first of these requirements (i.e., being depressed conveys some kind of survival and/or reproductive benefit to the depressed person), although being depressed may have some immediate beneficial outcome for the depressed person (i.e., withdrawal from uncontrollable aversive environmental stressors, enlisting others' help in dealing with those stressors), it is difficult to immediately see how being depressed conveys an advantage to the depressed person in an evolutionary (i.e., reproductive fitness) sense. In order to meet this criteria, the evolutionary hypothesis would have to show that simply being depressed helped depressed individuals to obtain an advantage in reproducing themselves and bringing their own offspring to reproductive maturity. To the contrary, data regarding the career success of people with depression indicate that they are less productive at work [52] and earn between 13% and 18% less than their nondepressed colleagues [53], thus providing them with less of the income which may be used to provide for a family and for their own health and well-being. Further, 20% of depressed employees report complete or occasional inability to carry out work responsibilities, 83% say they lack motivation, and 82% report difficulties concentrating [54], further reducing their value to employers and thence their earning capacity. Depression may also prevent patients from earning at all, with 34% of unemployed people in the UK being excluded from work by depression [55]. In addition, being depressed increases the likelihood of suicide [56]. These are not the likely hallmarks of individuals who will produce successful offspring, at least within recorded history. Nor are the underlying behaviour patterns of these people likely to have been beneficial to their archeological ancestors, consisting as they do of low motivation to succeed, inability to perform tasks when under demand, and lack of the kinds of behaviours which lead to material wealth, health, and well-being. Even if being depressed interacted with a particular environmental situation and thereby provided a hypothetical advantage for the depressed person within such an environment, such an advantage has not been reported in the wider literature to date, apart from being difficult to envisage. In fact, and as mentioned above, depression's parallel risk status with smoking as a predictor of mortality (even when related health factors such as blood pressure, alcohol intake, cholesterol and social status are taken into account) argues against there being any demonstrated survival value for depression [11]. Thus, the likelihood of depression holding any (previously hidden) compensatory effects on survival remains to be demonstrated, at best. 

The second requirement of an evolutionary trait (i.e., the depressed characteristic is passed on to offspring genetically) is met in that family studies of depression reliably produce a figure of 80% heritability [57, 58] for bipolar disorder, with some data identifying specific alleles that are responsible for parent-child transmission [59]. Similar data have been reported for parent-child links for Major Depressive Disorder (MDD), with children whose both parents had MDD having a 74% chance of developing the same disorder and children with one parent with MDD having a 27% chance of becoming severely depressed compared to 7% for children with neither parent meeting the criteria for MDD [60]. Other studies (e.g., [61, 62]) reported similar results, suggesting that having parents who suffered from depression was a strong indicator of elevated risk of offspring also developing depression. In addition to the genetic linkage studies reported earlier in this paper, these data support the presence of a genetic inheritance causal variable in transmission of depression; children of depressed parents are also more likely to be depressed themselves than children of nondepressed parents. 

Third (i.e., being the child of depressed parents enhances a child's own survival and reproductive success), there are multiple studies which have reported that children of depressed parents react more negatively to stressors, are delayed in their acquisition of self-regulation strategies, have more school problems, higher levels of behaviour problems and lower self-esteem (for reviews, see, [63–65]). Even though the construct of antagonistic pleiotropy (i.e., that a gene may transmit beneficial as well as damaging traits: [66]) might be raised as a possible way in which depression holds an evolutionary advantage for the later offspring of depressed parents (i.e., that the benefit might not emerge in the first generation of the depressed parents' offspring but may be apparent in later generations), there are no data extant in the literature which support this hypothesis. To the contrary, as well as the presence of disorders in children of depressed parents including anxiety [67], many adjustment problems (mentioned above) at school and later at work [68], substance abuse [69], and relationship difficulties [70], the incidence of those disorders among children is very large. For example, parental MDD is associated with an eightfold increased risk of MDD in their children, a threefold increased risk of anxiety disorders, and a fivefold increased risk of conduct disorder [71]. In addition, the presence of these problems has been noted in grandchildren of depressed parents [72], arguing against the antagonistic pleiotropy hypothesis in this case. Further, in a study of the effects of pleiotropy in the genes responsible for depression, Gorwood [73] reported that the strongest genetic link with MDD was generalized anxiety disorder. While there may be some epigenetic influences present in these behaviours that inculcate parental behaviour in the development of children's predisposition to depression [74, 75], the surmise that these children are likely to succeed at school, become career successful, and amass the large resources that are characteristics of individuals whose offspring have the greatest chance of reproducing themselves and managing their lives effectively (i.e., show reproductive fitness) is not supported by fact [61]. Again, the kinds of behaviours shown by children of depressed parents and listed above would not be likely to confer a reproductive advantage for the ancestors of *H. sapiens.*

### 2.2. Adaptive

However, the “evolutionary” argument might be modified to explain one set of *immediate* benefits of depressive behaviour to those individuals who suffer from the disorder without necessary reference to the reproductive success of their offspring. That is, by reference to the argument underlying the “evolutionary” hypothesis that being depressed provides *an adaptive advantage for the individual in the current environment*. As mentioned briefly above, one theory of depression which is based upon the assumption that depressive behaviour bestows some benefit upon the person exhibiting it has been described by several authors (e.g., [76–78]). As noted by Bolling et al. [32], “Identified features of the depressive syndrome are all in the spectrum of a withdrawal from contact with the world and the consequences of activity” (page 127). Applied to the specific symptomatology of depression, sadness, anhedonia, sleep and appetite change and cognitive disturbances are part of a “biological response pattern … identified as the conservation-withdrawal response to excesses or deficits of stimulation” (page 127) that includes low self-esteem, hopelessness, and helplessness. This withdrawal from aversive stimuli and environments has been described as “adaptive” in that it reduces the quantum of noxious stimuli to which the person is exposed [35, 36]. An important aspect of the mechanism underlying this withdrawal is the conviction on the part of the depressive person that he/she has no real control over the unpleasant experience that is occurring [35, 36] and therefore is left with a single response that will reduce distress—withdrawal from the environment that is causing the unpleasantness. 

This immediate benefit from depression is what Darwin 1876/2002 ([79, page 51]) referred to when he commented that “But pain or suffering of any kind, if long continued, causes depression and lessens the power of action; yet it is well adapted to make a creature guard itself against any great or sudden evil”. That is, while depression may provide an advantage of some degree to the sufferer, that advantage may not be of major benefit and certainly does not enhance the likelihood of reproducing successfully. In addition, maintenance of depressive withdrawal behaviour means that the depressed individual also loses access to available sources of social reinforcement, potentially setting up a circular process in which withdrawal from an aversive environment becomes both self-reinforcing and self-defeating [78]. As such, while depressive responses to unmanageable stressors may be initially “adaptive”, they probably are not so in the longer term. Furthermore, depressive withdrawal does not need to be “evolutionary” (i.e., transgenerational) in order to be conceptualized as conveying some *immediate* benefit to the depressed individual. That is, depressive may simply be “adaptive” in the short term. This leaves the investigation of depression open to consideration of the immediate “adaptive” benefits of depressive-withdrawal responses to unmanageable stressors.

## 3. Implications for Research

Because depressive withdrawal may convey some short-term advantages to the depressed individual, investigation of these advantages, and the environmental stressors which cause the individual to withdraw, presents a potentially fruitful area of research. If it is assumed that (according to the adaptation model of depressive withdrawal behaviour described above) the major advantage which proceeds from withdrawal is the relative absence of the stressor, then it becomes even more relevant to investigate the kinds of stressors which the depressed individual seeks to escape from when they withdraw. In their examination of the kinds of stressors that were associated with depression, Keller and Nesse [80] investigated the match between categories of stressors which they termed “precipitants” (page 27) and the presence of six subgroups of depressive symptoms assessed via the Centre for Epidemiological Studies Depression Scale. Different stressors were associated with different depressive symptoms: for example, social losses were associated with crying and arousal, and failure to reach a goal was linked with fatigue and pessimism. These findings were replicated [81] and extended to include nine sets of stressors [82]. However, the focus of those investigations was upon *categories *of stressors (such as death of a loved one, failure, romantic loss, inability to cope, social isolation and winter), and it may also be fruitful to investigate the *specific events* which individuals find sufficiently stressful to initiate withdrawal behaviour (and which, thereby, convey some advantage to the depressed individual). As has been shown previously, not all assumed stressors have negative effects upon those who experience them, and some even have reportedly positive effects [83, 84], thus challenging the validity of grouping many stressors into categories.

## 4. Clinical Directions

The focusing of research investigations onto the stressors which precipitate depressive withdrawal thus appears to most likely be of benefit if it is performed from an ideographic perspective rather than a nomothetic one. That is, identification of groups or categories of stressors may be valuable as a first step in this process, but clinical treatment models that are based upon an adaptive model of depression require more precise identification of those specific stressors which initiate depressive withdrawal behaviour in a particular patient. One model which proceeds on this basis is Functional Analysis of depression Kanter et al., [85]; Kohlenberg et al., [86]. This approach is well suited to clinical investigations of depression from an adaptive perspective because it assumes that the depressive behaviour itself is reinforced by its consequences, thus readily identifying the specific stressors, the depressive behaviour, and the advantages which flow to the depressed individual from their depressive (withdrawal) behaviour.

## 5. Conclusion

Initially, depression appears to confer such misery upon the sufferer that it may be difficult to imagine that it also has advantages for that person. However, like other physiological functions which cause discomfort (such as raised temperature and inflammation) depressive withdrawal behaviour may also have a functional aspect. As noted by Moskowitz [87], even the apparently bizarre behaviour of catatonia can have self-protective benefits for the organism (i.e., stillness in the face of unavoidable threat from predators can assist in evading death), and depressive withdrawal may also convey a similar benefit to the depressed person. While it cannot be classified as evolutionary because of the reasons given above, withdrawal from uncontrollable danger or pain is, from all perspectives, advantageous for the person who exhibits such behaviour and therefore may be seen as adaptive.
